# Prediction of Mechanical Properties of Fly-Ash/Slag-Based Geopolymer Concrete Using Ensemble and Non-Ensemble Machine-Learning Techniques

**DOI:** 10.3390/ma15103478

**Published:** 2022-05-12

**Authors:** Muhammad Nasir Amin, Kaffayatullah Khan, Muhammad Faisal Javed, Fahid Aslam, Muhammad Ghulam Qadir, Muhammad Iftikhar Faraz

**Affiliations:** 1 Department of Civil and Environmental Engineering, College of Engineering, King Faisal University, P.O. Box 380, Al-Ahsa 31982, Saudi Arabia; kkhan@kfu.edu.sa; 2 Department of Civil Engineering, Abbottabad Campus, COMSATS University Islamabad, Abbottabad 22060, Pakistan; arbabfaisal@cuiatd.edu.pk; 3 Department of Civil Engineering, College of Engineering in Al-Kharj, Prince Sattam Bin Abdulaziz University, Al-Kharj 11942, Saudi Arabia; f.aslam@psau.edu.sa; 4 Department of Environmental Sciences, Abbottabad Campus, COMSATS University Islamabad, Abbottabad 22060, Pakistan; hashir785@gmail.com; 5 Department of Mechanical Engineering, College of Engineering, King Faisal University, Al-Ahsa 31982, Saudi Arabia; mfaraz@kfu.edu.sa

**Keywords:** fly ash, slag, machine-learning, validation, parametric analysis, ensemble approaches

## Abstract

The emission of greenhouse gases and natural-resource depletion caused by the production of ordinary Portland cement (OPC) have a detrimental effect on the environment. Thus, an alternative means is required to produce eco-friendly concrete such as geopolymer concrete (GPC). However, GPC has a complex cementitious matrix and an ambiguous mix design. Aside from that, the composition and proportions of materials utilized may have an impact on the compressive strength. Similarly, the use of robust and efficient machine-learning (ML) approaches is now required to forecast the strength of such a composite cementitious matrix. As a result, this study anticipated the compressive strength of GPC with waste resources using ensemble and non-ensemble ML algorithms. This was accomplished through the use of Anaconda (Python). To build a strong ensemble learner by integrating weak learners, adaptive boosting, random forest (RF), and ensemble learner bagging were employed. Furthermore, ensemble learners were utilized on non-ensemble or weak learners, such as decision trees (DT) and support vector machines (SVM) via regression. The data encompassed 156 statistical samples in which nine variables, namely superplasticizer (kg/m^3^), fly ash (kg/m^3^), ground granulated blast-furnace slag (GGBS), temperature (°C), coarse and fine aggregate (kg/m^3^), sodium silicate (Na_2_SiO_3_), and sodium hydroxide (NaOH), were chosen to anticipate the results. Exploring it in depth, twenty sub-models with ensemble boosting and bagging approaches were trained, and tuning was performed to achieve the highest possible coefficient of determination (R^2^). Moreover, cross K-Fold validation analysis and statistical checks were performed via indicators for the evaluation of the models. The result revealed that ensemble approaches yielded robust performance compared to non-ensemble algorithms. Generally, an ensemble learner with the RF and bagging approach on a DT yielded robust performance by achieving a better R^2^ as 0.93, and with the lowest statistical errors. The communal model in artificial-intelligence analysis, on average, improved the accuracy of the model.

## 1. Introduction

The emissions of greenhouse gas (GHG) in the environment have caused the melting of glacier reservoirs, which tremendously contributes to major threats to the globe [1]. The concrete sector is believed to be the most significant source of greenhouse-gas emissions, contributing up to 50% of world emissions [2]. Thus, Portland cement (PC), an essential component of concrete, significantly contributes to GHG emissions [3]. The production of PC contributes around 7% to the atmosphere and the environment. Furthermore, the calcination of calcium oxide (CaO) during the cement-manufacturing process accounts for 50% of CO_2_ emissions [4]. Currently, 4000 million tons of PC are produced annually, with an anticipated of about 6000 million tons by 2060 [5]. These figures show the need for alternative measures to meet the rising demand for concrete while using fewer resources and effectively emitting less CO_2_ [6,7]. Therefore, the utilization of leftover recycled and waste substances in concrete is one of the proposed scientific and realistic remedies for reducing its high demand [8,9,10,11]. This will not only meet the growing need for concrete, but it will also reduce the risk to the environment [9]. In this regard, fly ash (FA) and GGBS as natural pozzolanic materials can be effectively used as supplemental cementitious materials in the construction sector [12,13,14,15]. Thus, their use in the building sector could reduce the environmental consequences associated with the manufacturing and usage of cement in the building industry. Moreover, the addition of these materials with alkaline solvents such as Sodium silicate (Na_2_SiO_3_) and sodium hydroxide (NaOH) produces viable and eco-friendly environmental concrete such as geopolymer concrete (GPC) [16,17,18,19]. The amorphous gel form of GPC possesses many outstanding and attractive characteristics, including resistance to sulfate attack, acid resistance, enhanced durability, fire resistance, and an undoubtedly greater compressive strength than conventional concrete [12,20,21,22,23]. Likewise, their use in the construction industry can extensively lessen CO_2_ emissions in the atmosphere [24]. Moreover, the difference between ordinary Portland cement (OPC) and GPC is illustrated in Table 1. Studies have revealed that the chemical and physical properties of the matrix have a major influence on the strength of GPC. Thus, the fly-ash-to-NaOH ratio, Na_2_SiO_3_-to-NaOH ratio, workability, fly-ash-to-sand ratio, molarity, and alkaline ratio affect the strength of concrete [25,26,27]. Ukritnukun et al. [28] observed that the blast-furnace slag concentration, curing temperature, and silicate modulus all had a beneficial effect. Additionally, Asghar et al. [29] determined the ideal molar ratios of Ca/Si (calcium oxide/silica) and (Na + K)/Si ((sodium + potassium)/silica), as well as the ideal volume ratio (H_2_O/solid)_vol_ for increasing the strength properties of GPC. Songpiriyakij et al. [30] found that a Si-to-Al ratio of 15.9 resulted in the formation of GPC with the relatively high compressive strength of 73 MPa. Puertas et al. [31] examined the strength and growth characteristics of fly-ash/slag-paste-hydration products. After 28 days of curing at 25 °C, they reported that the mechanical properties of the mix with a fly-ash/slag ratio of 1.0 that was cured at 25 °C and stimulated with a 10 M NaOH solution exceeded 50 MPa. Moreover, according to Rai et al. [31], the cumulative effect of NaOH molarity, curing temperature, and activator-to-binder ratio directly impacts the initial compressive strength, while the NaOH/Na_2_SiO_3_ ratio is not statically important, and the target strength can be attained more quickly at high temperatures than at room temperatures.

To make GPC, pozzolanic materials with binding properties are polymerized at high temperatures in an alkaline medium [39]. As a result, a crystalline and amorphous compound is formed, which can be used to achieve the desired mechanical properties [39]. However, the high demand for heat curing in the production of a geopolymerization compound is not recommended for in-field application. Due to the high heat demand of curing, this will limit the use of FA-GPC in the construction domain [40]. Thus, heat demand can be reduced by using a slag blend with a high concentration of calcium, silica, and alumina. The use of the GGBS slag blend in conjunction with FA gives a dense microstructure with hydrated and polymerization products that significantly improve the early age strength of GPC. Yazdi et al. [41] examined the outcome of GPC by varying the dosage of FA with GGBS. The author showed that replacing FA with GGBS results in a significant increase in compressive and flexural strength of 100 MPa and 10 MPa, respectively. Furthermore, Fang et al. [40] studied the varying dosage of slag content on the flexural and split tensile strength of FA-GPC. The author revealed a higher strength due to the formation of C-A-S-H gel and N-A-S-H. This ultimately speed up the reaction process of GPC [40]. The compressive strength of concrete is typically evaluated by conducting physical tests. In general, concrete specimens that are cubical and cylindrical in shape are produced by using precise mixture ratios and curing with water for approximately 28 days to yield the hydrated products [42]. Afterwards, the compressive strength is determined using a compression-testing machine. This approach is common in the execution of work in the field and laboratory, yet it is inefficient and time-consuming. Rather than using standard experimental procedures to determine the compressive strength of concrete, empirical regression methodologies are preferable for estimating the strength of concrete [43]. On the other hand, the literature reveals that the chemical composition and physical proportions of variables have a significant impact on the GPC [44]. Moreover, heterogeneity exists in the production of GPC as a result of the variety of parameters involved. While various algorithms and methods based on statistical approaches are capable of evaluating the compressive nature of GPC, the relationship between factors and mechanical strength is not well understood. Thus, machine-learning (ML) approaches may now be used to predict the compressive strength of concrete, thanks to recent advances in artificial-intelligence algorithms [45,46,47,48,49,50,51,52]. The evolution of the advanced prediction algorithms could be used for a variety of purposes, such as regression, classification, and clustering of data [53]. Estimating the compressive loading capacity of concrete is just one application of the ML regression function. The ML methodology, in contrast to prior regression methods, delivers very precise results [54,55]. The discovery of artificial-intelligence algorithms such as genetic engineering programming (GEP), support vector machine (SVM), artificial neural network (ANN), and ensemble approaches has enabled researchers to address tough problems [56,57,58,59,60,61].

This research will investigate the effect of network- and tree-based models for prediction by employing boosting, AdaBoost (bagging), and utilizing modified bagging random forest (RF). Unlike previous research, this study does not exclusively depend on ensemble techniques, but also discusses the tree- and network-based studies on ensemble learning. Second, this study is based on modeling of ensembles over individual models in order to anticipate the compressive behavior of GPC using secondary raw materials. To the authors’ knowledge, no work similar to ensemble ML models for GPC has been employed. Furthermore, this modeling was carried out in Anaconda navigator version 1.9.12 with Python version 3.7.

## 2. Database Presentation Using Python

**Figure 1:**
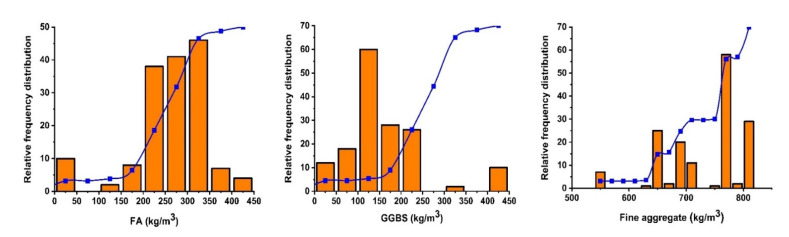

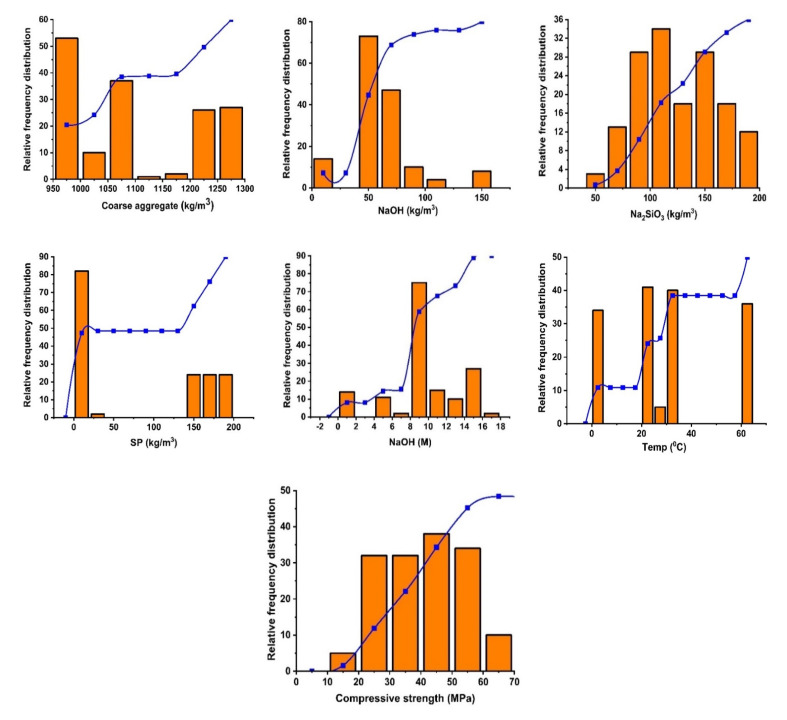
Frequency distribution of input and output parameters.

For the representation of the database, the Anaconda-based Python programming (version 3.7) was utilized from the published literature (Table S1) [62,63,64,65,66,67,68,69,70,71,72]. The data were gathered from the accessible literature and comprise nine parameters, namely as fly ash (kg/m^3^), alkaline activator (kg/m^3^), aggregate (kg/m^3^), GGBS (kg/m^3^), NaOH molarity, SP dosage (kg/m^3^), curing temperature (°C) and an output parameter of compressive strength as illustrated in Figure 1. Every parameter that was chosen had a significant impact on the strength qualities of fly-ash-slag-based concrete. Moreover, the Python programming language was used to find the link between these variables and concrete compressive strength. Additionally, the influential variables in forecasting the mechanical strength were evaluated through the use of permutation features. Furthermore, Table 2 illustrates the variable range values with maxima and minima based on the 156 data points, while Table 3 displays the results of the statistical-analysis check, which includes the mean, the count, and the standard deviation. The parameters used in making the models have a substantial influence on the model’s robustness. Seaborn, a command in Python, is used to employ machine learning (ML) and to depict the correlation between two variables.

## 3. Methods

**Figure 2:**
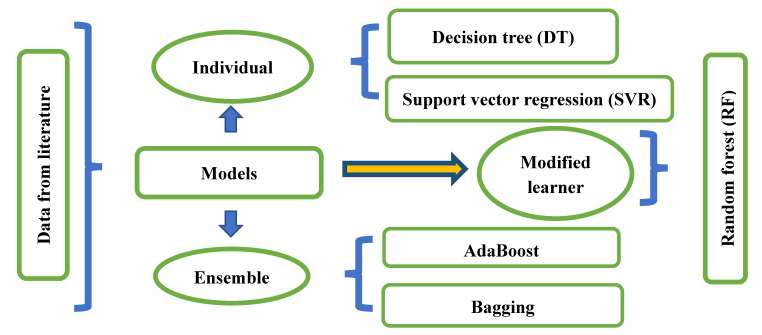
Flow diagram of models used in this research.

ML technologies are now being used in a wide range of industries to anticipate and understand the nature of various constituents. In this study, ML-based methods such as SVM, the decision tree (DT), RF, and multiple linear regressions (MLR), were utilized to estimate the compressive strength of GPC. These methods were chosen for their popularity, robustness in predicting outcomes, and were recognized as the top evaluated algorithms. Furthermore, the ensemble model with weak learners was utilized to model the strength of GPC utilizing AdaBoost and bagging. Moreover, Figure 2 depicts the entire systematic diagram of the individual and ensemble learning approach.

### 3.1. Decision Tree

This is a supervised ML approach that creates a tree-like model from training data using the DT. It is similar to a schematic flow in that each of the vertices reflects a test of a characteristic and that each route reflects the result of the test o that feature. It is referred to as a DT due to the fact that its form is comparable to that of a tree. This is accomplished through the use of partitions in predictors, which allows the target variables to be based primarily on divisions between the input parameters. Due to the fact that the regression tree automatically picks values, the educated regression tree presents parameters that are much more relevant to anticipate target variables from the preceding tree node than variables, which are less important to predict target variables. Because the specified dataset has no classifications, a regression model is fitted to the target variable using the independent variables. Every variable has several sites of division. The technique compares the predicted and actual numbers for each division point. The split point errors for all variables are summed, and the variable with the fitness function’s fewest values is chosen as the split point. This process is repeated.

### 3.2. Random Forest

The RF approach is both a regression and a cataloging approach, and it has been the subject of the majority of the research work. Breiman invented RF regression in 2001, and it is widely regarded as an improvement over traditional classification regression methods. It is reported that the key advantages of RF are its flexibility and speed in building input–output relationships. The main difference between DT and RF is that DT only builds one tree whereas RF builds a forest of trees where dissimilar data are randomly picked and given to each tree. The data are organized into rows and columns for each model tree, with different sizes of columns and rows being used for different trees. Moreover, the development of every tree is carried out in the sequence of phases shown below.

1. Approximately two-thirds of the entire dataset is picked at random for each forest and is symbolized by the data frame, a process known as bagging. In order to discover the optimum node-splitting technique, the predictor parameters are chosen at random.
2. Out-of-bag error is assessed for all of the trees based on the data that are available. Then, the mistakes from each tree are added together in order to yield the final output for each tree.
3. Each tree provides a statistical analysis based on regression, and the algorithm chooses the forest that receives the greatest number of votes. The votes might be 0 or 1. The fraction of 1 s is a prediction probability.

### 3.3. Support Vector Regression

Vapnik is considered to be the originator of SVM, which was initially utilized in the year 1995. It is now frequently used for classification, prediction, and regression. Because SVMs can effectively handle nonlinear regression problems, they are commonly utilized in input–output analysis. This is accomplished by applying a static diagraming strategy to the SVM analysis data in order to map them into n-dimensional function space. After that, the nonlinear activation operations are employed to match the substantially high-dimensioned space in which the information on the input parameters is more distinct from the original data, leading to a much more precise match. The linear function in space is denoted by the symbol *f* (*x*, *w*), which may be written as follows:

(1) $$f\left(x,w\right)=\sum_{j=1}^{n}w_{j}g_{j}\left(x\right)+b$$

where, ‘*b*’, ‘*g_j_*(*x*)’, and ‘*w*’ denote the nonlinear bias term, input space, and weight vector transformations determined by enhancing the normalized risk function, respectively. Assessment quality is also calculated by a loss function *L_ε_*, where *L_ε_* can be given as follows.

(2) $$L_{\varepsilon }=L_{\varepsilon }\left(y,f\;\left(x,w\right)\right)=\{\;\begin{array}{c} 0\\ \left|y-f\left(x,w\right)\right| \end{array}\;\;\;\;\begin{array}{c} if\;|y-f\left(x,w\right)\leq \;\varepsilon \;\\ \mathrm{otherwise} \end{array}\;\;\;\;$$

SVM regression is unique in that it uses an ε-insensitive loss function to compute a linear regression function for the additional higher-dimensional space while minimizing model complexity ${\left|\left|w\right|\right|}^{2}$. This job is proven by non-negative slack variables *ξ_i_* + ξ_i_^*^, where *I* = 1, …, *n* is used to find models from the π-insensitive field. Thus, the SVM regression can be built by streamlining the function as follows:

(3) $$\min\frac{1}{2}\;{\left|\left|w\right|\right|}^{2}+C\sum_{i=1}^{n}\left(\xi_{i}+\xi_{i}^{*}\right)$$

(4) $$\mathrm{subject}\;\mathrm{to}\;\{\begin{array}{c} y_{i}-f\left(x_{i},w\right)\leq \varepsilon +\xi_{i}^{*}\\ f\left(x_{i},w\right)-y_{i}\leq \varepsilon +\xi_{i}^{*}\\ \xi_{i},\xi_{i}^{*}\geq 0,\;i=1,\ldots ,n \end{array}$$

This optimization issue may be turned into a dual situation that can be resolved by

(5) $$f\left(x\right)=\sum_{i=1}^{nsv}\left(\alpha_{i}+\alpha_{i}^{*}\right)K\left(x,x_{i}\right)\;\mathrm{subject}\;\mathrm{to}\;0\leq \alpha_{i}^{*}\leq C,0\leq \alpha_{i}\leq C$$

where *n_SV_* is the quantity of provision vectors. The kernel function is

(6) $$K\left(x,x_{i}\right)=\sum_{i=1}^{m}\left(g_{i}\left(x\right)+g_{i}\left(x_{i}\right)\right)\;$$

During the training process, selected SVM kernel functions such as the linear, radial basis, polynomial, and sigmoid functions are used to determine support vectors along the function surface of the function surface. The kernel settings are influenced by the type of kernel used and the software that is implemented.

### 3.4. Boosting and Bagging Ensemble Approaches

**Figure 3:**
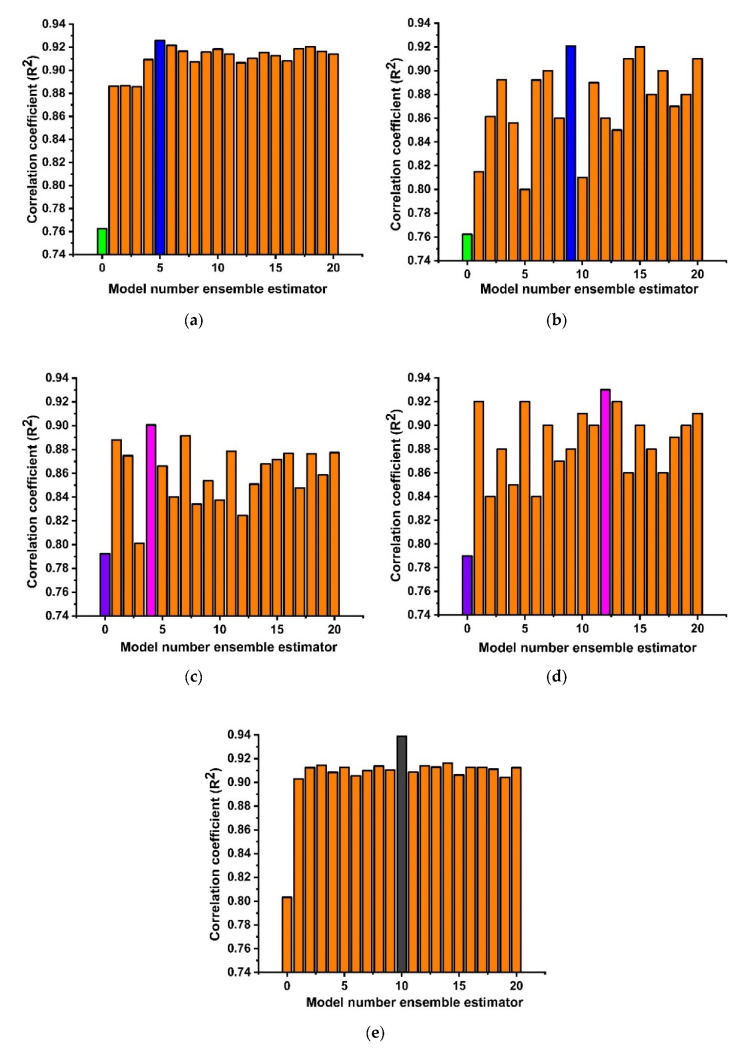
Ensemble modeling (**a**) DT with AdaBoost algorithm; (**b**) DT with bagging algorithm; (**c**) SVR with AdaBoost algorithm; (**d**) SVR with bagging algorithm; (**e**) RF ensembling.

Ensemble techniques are used to improve ML recognition and prediction accuracy. By integrating and aggregating numerous weaker prediction models, these methods generally assist in alleviating over-fitting issues (component sub-models). It is possible to make a smarter learner by intelligently altering training data and constructing several sub-models (A, B, …, N). Furthermore, the ideal model may be made by merging prominent sub-models using voting and averaging combination measures to reach the best possible result, as illustrated in Figure 3. Bagging is among the most widely used ensemble modeling techniques, which uses the bootstrap resampling method to gather data and calculate benefits. During the bagging procedure, the first training set substitutes partial models from the actual model. A few data samples can appear in multiple models, whilst some do not appear at all in any product models. The final model outcome is then calculated by taking an average of the outputs from all of the component models.

The boosting process, like the bagging technique, generates a cumulative model that results in the construction of a number of components that are more precise than non-ensemble models. Additionally, boosting is the process of using weighted averages by relying on sub-models to determine where it should be included in the finalized model. Based on individual learners such as SVM, DT, and RT, this study predicts the strength of GPC using boosting and bagging techniques.

There are two types of tuning parameters utilized in communal (ensemble) algorithms: (i) parameters that are connected with the perfect amount of model learners, and (ii) learning rates. The boosting and bagging algorithms with twenty ensemble models were made from the individual base learner and the best model constructs were picked based on strong correlation coefficient values, as shown in Figure 3 and Table 4. It can be seen that the DT with AdaBoost and bagging with N = 5 and 9 yields an R^2^ of 0.92. Moreover, support vector regression (SVR) shows a similar trend with an estimator of 4 and 12 yielding a strong correlation of about 0.90 and 0.93, respectively.

## 4. Model Assessment Using Statistical Measures

The robustness of the model is evaluated by statistical checks in the form of error measures for individual and ensemble models are presented from Equations (7) and (8)

(7) $$\mathrm{MAE}=\frac{1}{n}\sum_{i=1}^{n}\left|x_{i}-x\right|$$

(8) $$\mathrm{RMSE}=\sqrt{\sum \frac{{\left(y_{pred}-y_{ref}\right)}^{2}}{N}}$$

## 5. Result

**Figure 4:**
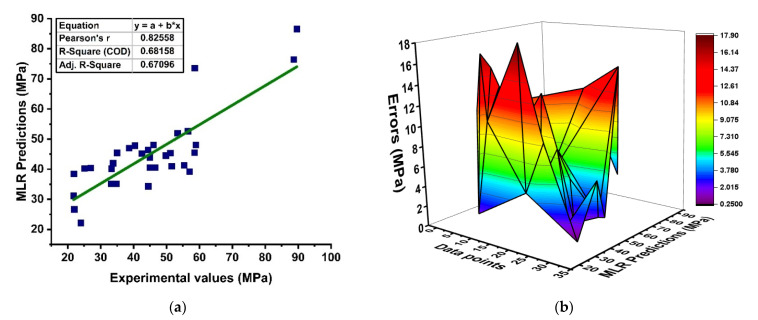
(**a**) Linear regression modeling; (**b**) distribution of errors via regression.

A linear regression model for predicting GPC with variable influences is illustrated in Figure 4. It should be noted that the Python-based approach has a strong correlation in the prediction of strength, as demonstrated in Figure 4a. However, this approach shows a lesser correlation in prediction with R^2^ = 0.637. In addition, the difference between the prediction and target in terms of its absolute-error distribution is illustrated in Figure 4b, showing that the majority of the predicted outcomes depict greater error with 17.87 MPa (maximum), 0.29 MPa (minimum), and 7.69 MPa (average) absolute error, specifying that the data set of the model is biased. It shows that linear regression may be used to anticipate non-linear analysis results to a limited extent. Although, the MLR model cannot be used for non-linear analysis outcomes that have the strongest correlation to their outcome.

### 5.1. Decision Tree

**Figure 5:**
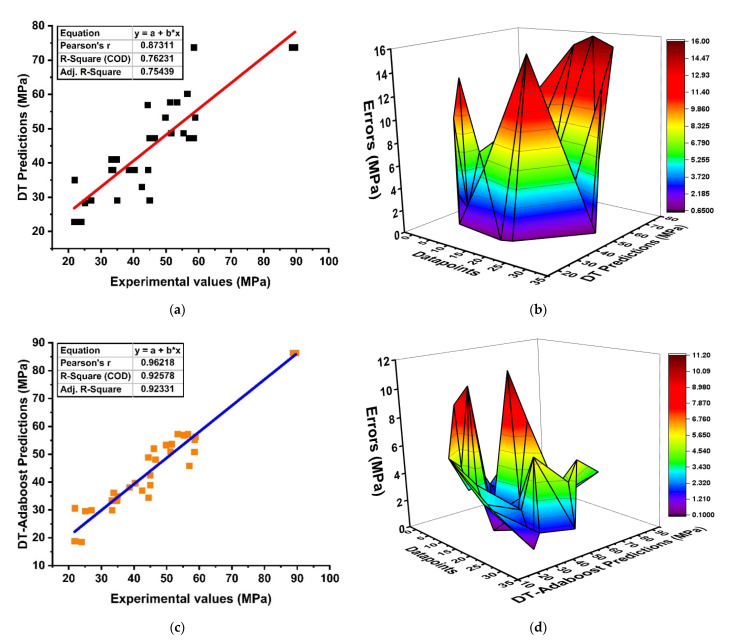

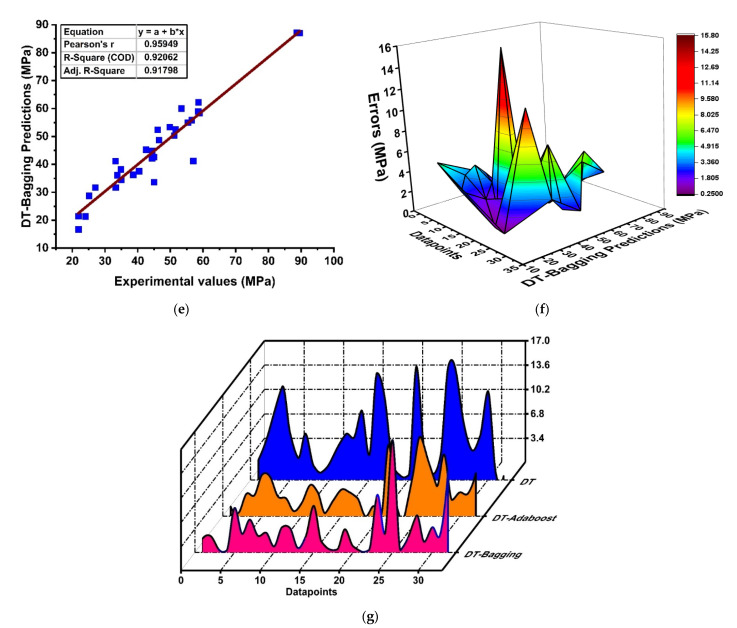
DT modeling; (**a**) non-ensemble model; (**b**) non-ensemble absolute-error distribution; (**c**) ensemble model of DT with boosting approach; (**d**) absolute errors of DT with boosting approach; (**e**) ensemble model of DT with bagging approach; (**f**) absolute errors of DT with bagging approach; (**g**) Comparison of models with errors using waterfall plot.

The supervised and nonlinear regression model with a DT provided a soundly favorable prediction outcome, as depicted in Figure 5. In addition, the DT was modeled using several ensemble methods, such as bagging and boosting, as depicted in Figure 5. It can be seen in Figure 5a that the DT as an individual algorithm produces a good relationship with R^2^ = 0.76. Moreover, the performance of the model can also be assessed by its absolute error, as demonstrated in Figure 5b. However, the model accuracy and outcome prediction can also be evolved by using ensemble approaches due to its performances and robustness. In addition, adding a boosting regressor to the weak or individual learner shows a positive correlation with R^2^ = 0.92, as depicted in Figure 5c, with its reduced error distribution in Figure 5d. The bagging model illustrates a good R^2^ = 0.92 with average errors of 15.78 MPa (lesser maximum), 0.26 MPa (minimum), and 3.22 MPa compared to MLR, as shown in Figure 5e,f. Although, the same individual model was modeled with AdaBoost regressor, showing a clear significant enhancement of the model. Moreover, the efficiency of the model can also be judged by its absolute errors, as depicted in Figure 5g. Its shows that the model performance is significantly enhanced as compared to the MLR model.

### 5.2. Support Vector Regression

**Figure 6:**
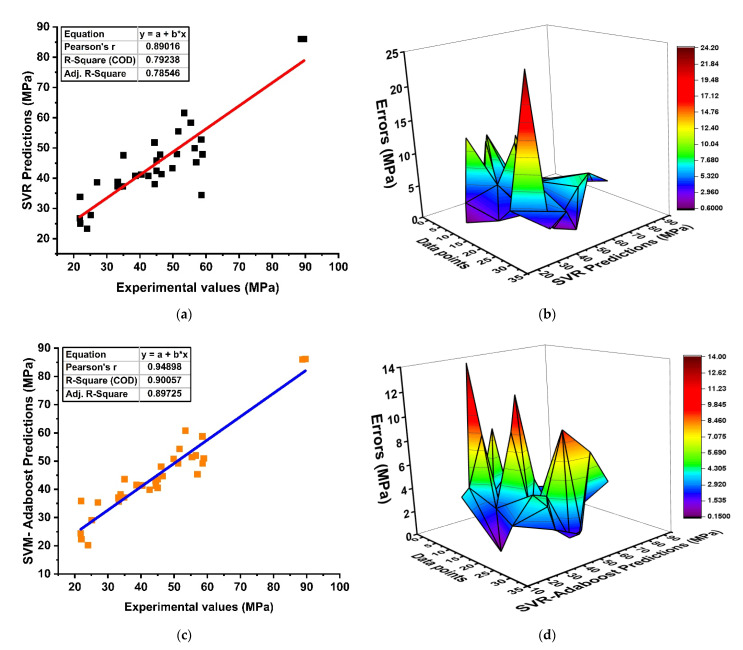

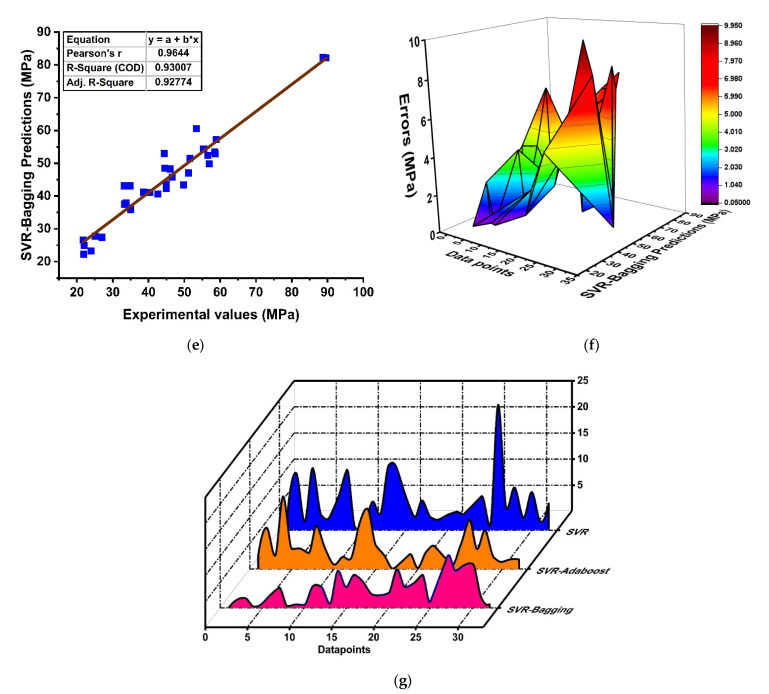
SVR modeling; (**a**) non-ensemble model; (**b**) non-ensemble absolute errors distribution; (**c**) ensemble model of SVR with boosting approach; (**d**) absolute errors of SVR with boosting approach; (**e**) ensemble model of SVR with bagging approach; (**f**) absolute errors of SVR with bagging approach; (**g**) Comparison of models with errors using waterfall plot.

ML with SVR was carried out to predict the mechanical properties of GPC, as shown in Figure 6. The predicted outcome with experimental data points as individual regression models depicts a strong relationship with R^2^= 0.79 due to its obstinate generalization capacity in making a robust performance, as shown in Figure 6a. Similarly, to the DT, SVR model accuracy can also be evaluated by its absolute-error distributions, as depicted in Figure 6b. It shows that the overall results of the predicted outcome lie close to the experimental values with minor data lying as outliers, but it does not devalue the accuracy of the model. In addition, in terms of statistical measures, SVM models show reduced average errors of about 5.69 MPa as compared to MLR (7.69 MPa). Likewise, the SVM model is ensembled and thus shows significant enhancements as depicted in Figure 6c,e with R^2^ = 0.90 and R^2^ = 0.93, respectively. Figure 6c,d represent the regression analysis of the boosting algorithm with its error distribution, showing that the boosting algorithm has an obstinate effect on forecasting the properties of concrete. Overall, the efficiency of the model can also be evaluated by its maximum (13.97 MPa), minimum (0.19 MPa), and average errors (4.14 MPa), and it is reported as a minimum compared to MLR. In addition, the bagging algorithm shows a similar trend by yielding a reasonable model with R^2^= 0.93 and its error distribution of 9.92 MPa (maximum), 0.08 MPa (minimum), and 3.76 MPa (average), as illustrated in Figure 6e,f. The overall comparison between SVR and bagging and boosting in terms of their absolute errors is shown in Figure 6g. The model with SVR demonstrates a significant and accurate prediction due to the strong learner in the model.

### 5.3. Random Forest

**Figure 7:**
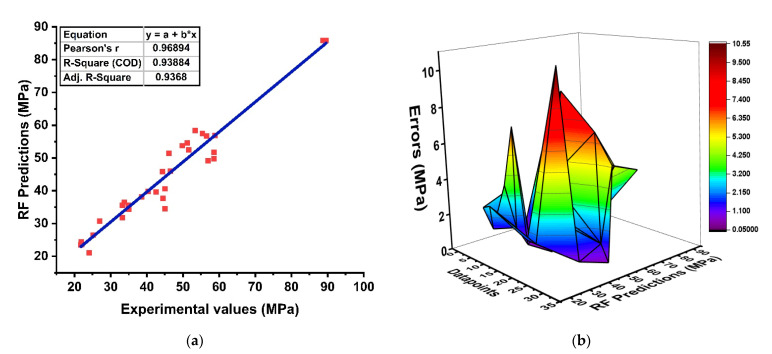
RF; (**a**) Relationship of experimental and predicted values; (**b**) Error-distribution result of model.

The RF algorithm is a type of ensemble ML approach that incorporates the bagging method and random-feature-selection procedure to yield a predictive model. The predictive performance between the target and experimental results is depicted in Figure 7. The model illustrates a well-defined correlation with R^2^= 0.938 and is also assessed by its absolute error distributions as illustrated in Figure 7b. It can be seen that the RF-based model gives a lesser difference between prediction and experimental values with maximum, minimum, and average errors of about 10.54 MPa, 0.08 MPa and 3.217 MPa, respectively. Similarly, the forecasted results show that the influence of the strong learner in prediction is far better than individual approaches.

### 5.4. Cross-Validation Results

**Figure 8:**
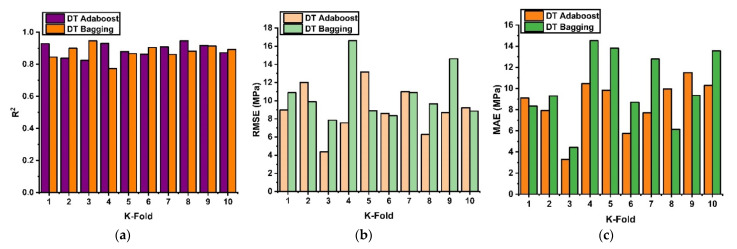

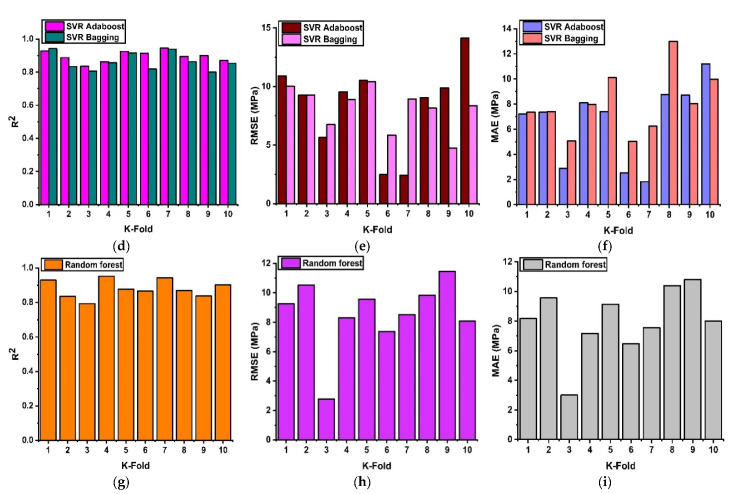
K-fold validations; (**a**) DT correlation with ensemble models; (**b**) DT RMSE errors with ensemble models; (**c**) DT MAE errors with ensemble models; (**d**) SVR correlation with ensemble models; (**e**) SVR RMSE errors with ensemble models; (**f**) SVR MAE errors with ensemble models; (**g**) RF correlation with ensemble models; (**h**) RF RMSE errors with ensemble models; (**i**) RF MAE errors with ensemble models.

In order to assess a model, it must have the desired level of accuracy. To assure the accuracy of prediction models, it is necessary to perform this validation. The validation of this model was performed by using a ten-fold validation, as illustrated in Figure 8. This strategy is intended to limit the degree of bias involved in selecting the training data set at random during the training process throughout the training phase. It divides the data that are used to make the model into ten equal sections.

It uses nine out of ten subsets to design the robust learner and one set to authenticate the model. This approach yields an average error accuracy and is evaluated through statistical errors. The ten-fold cross-validation approach is said to demonstrate the generalization and dependability of the model performance, as demonstrated in Figure 8. The DT model with the ensemble approach via AdaBoost and bagging depicts good ten-fold R^2^ values with an average values of R^2^ = 0.89 and 0.879 for the AdaBoost and bagging approaches, as illustrated in Figure 8a. Similarly, the model shows a significant validation response by showing lesser RMSE and MAE errors with 8.99 MPa and 10.65 MPa for both ensemble models, respectively, as shown in Figure 8b,c. Moreover, the validation response via the SVR model in terms of R^2^ shows an average error of 0.89 and 0.86 for the tenth k series for both models, as illustrated in Figure 8d. This depicts a strong accuracy of the models towards predictions. Likewise, the validation response in term of RMSE and MAE for the SVR model demonstrate the same response as for DT by showing lesser errors, as illustrated in Figure 8e,f. Additionally, the RF model depicts a comparable response to DT and SVR by adamantly representing a positive R^2^ relation with predicted values and showing lesser errors.

### 5.5. Statistical Analysis of Models

The evaluation of the models is also performed by conducting statistical measures. Apart from R^2^, the statistical check is significantly useful in the assessment of any model by measuring the numerical values, as depicted in Table 5. It can be seen that the individual model yields an MAE error of about 7.69 MPa, which is more than the ensemble models. DT with AdaBoost and bagging yields 53.3% and 58.12% more accurate models as compared to the individual. Similarly, RMSE and MSE show the similar response for the DT model. The SVR model shows that the ensemble model increases the efficiency of the models by 27.24%, 49.51%, and 28.99% for the AdaBoost model and by 33.92%, 60.8%, and 37.4% for the bagging model due to the incorporation of the weak learner in the making of a resilient model. Likewise, the RF model demonstrates a more efficient prediction model due to its lesser errors, as illustrated in Figure 7.

### 5.6. Permutation Features Analysis of Variables in Geopolymer Concrete

**Figure 9:**
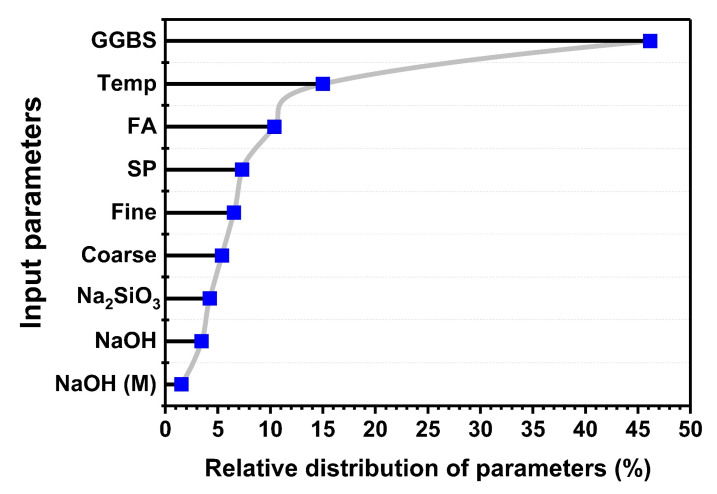
Permutation analysis of parameters in geopolymer concrete (GPC).

The permutation analysis depicts the influence of each variable on the target strength of GPC, and was conducted through the spyder notebook by using Python language in Anaconda software, as illustrated in Figure 9. The analysis results reveal that the GGBS, FA, and temperature (°C) have a significant effect on the strength of GPC due to the occurrence of major SiO_2_, Al_2_O_3_, and CaO in the amorphous state [73,74,75]. Additionally, the presence of GGBS in concrete gives rise to binding phenomena in the presence of the alkaline medium. Moreover, when GGBS is combined with FA in an alkaline medium, it gives rise to additional calcium content that is responsible for the enhanced mechanical properties.

## 6. Conclusions

The aim of this research was to anticipate the strength of GPC using the individual and ensemble ML approaches. For prediction, two individual approaches, DT and SVR, and three ensemble techniques, bagging, AdaBoost, and RF regression were used, and the following conclusions are drawn from the analysis.

1. The DT as an individual approach yields a positive outcome with R^2^ = 0.76. Nevertheless, the ensemble approaches with bagging and boosting depict precise results with R^2^ = 0.92. These indications make it clear that the ensemble approach yields positive results due to its weak-learner incorporation.
2. SVR shows a similar response with ensemble approaches as compared to the individual approach. Moreover, the SVR model shows superior performance by depicting a good coefficient of determination with R^2^ = 0.90 for boosting and R^2^= 0.93 for bagging. Similarly, RFR yields better performance with R^2^ = 0.93 for the testing set. This shows that the ensemble model yields robust performance as compared to non-ensemble approaches.
3. Cross-validation of the test set reveals lesser MAE, RMSE errors, and good average correlations of R^2^ for the DT, SVR, and RF, indicating the accuracy of the model. Statistical-analysis results reveal lesser error for MAE, RMSE and MSE as compared to individual approaches.
4. The RF and SVR with bagging were superior to individual and ensemble approaches by showing R^2^ = 0.93.
5. Permutation analysis of variables shows that FA, GGBS, and temperature have a major influence on the strength of GPC.

## Figures and Tables

**Table 1:** Comparison of GPC with OPC.

Attributes	GPC	OPC	Summary	References
Tensile strength	Greater	Lower	GPC has higher strength due to presence of aluminosilicate, activators and types of activators that enhance the strength at early age.	[32]
Acid attack	More resistance	Less resistance	Presence of aluminosilicate, activators and types of activators show enhanced resistance to acidic attack	[33]
Durability	More resistance	Less resistance	Presence of aluminosilicate, activators and types of activators show enhanced resistance to acidic attack	[34]
Compressive strength	Higher	Lower	Same factors as tensile strength	[35]
Porosity	Significantly less	Moderate	Internal geopolymeric structure and presence of aluminosilicate, activators and types of activators influence GPC porosity.	[36]
Fire resistance	Significantly higher	Limited	GPC concrete is more resistant to deterioration caused by high temperatures as compared to OPC.	[37]
CO_2_ emission	Lower	Higher	Utilization of waste materials shows lesser CO_2_ as compared to OPC	[38]

**Table 2:** Contribution of parameters with ranges.

Variables Used	Acronym	Minima	Maxima
Input variables			
Fly ash	FA	0	400
Fine aggregate	FIA	547	810.6
Ground granulated blast furnace slag	GGBS	0	409
Coarse aggregate	CAA	966	1293
Sodium hydroxide	NaOH	9	143.3
Sodium silicate	Na_2_SiO_3_	54	192.9
Super plasticizer	SP	0	180
Temperature	T °C	0	60
Output			
Compressive strength	*f_c_^’^*	10.5	89.6

**Table 3:** Descriptive data of parameters.

Statistical Description	FA	GGBS	Fine	Coarse	NaOH	Na_2_SiO_3_	SP	NaOH	Temp.
Mean	252.5	151.4	729.8	1096.0	60.5	123.0	77.6	8.6	28.1
Standard Error	6.9	6.9	5.4	9.4	2.1	2.9	6.5	0.3	1.6
Median	270.0	135.0	760.5	1090.8	57.1	115.7	7.9	8.0	25.0
Mode	303.8	101.3	774.0	1090.8	81.0	81.0	0.0	8.0	30.0
Standard Deviation	86.3	86.7	68.0	117.9	26.8	35.7	81.0	3.9	20.6
Sample Variance	7442.7	7522.7	4620.5	13,889.3	720.4	1275.1	6558.3	15.2	422.4
Kurtosis	2.5	2.2	0.0	−1.5	3.0	−0.9	−1.9	0.2	−0.9
Skewness	−1.4	1.3	−0.8	0.3	1.2	0.1	0.2	−0.5	0.3
Range	400.0	409.0	263.6	327.0	134.3	138.9	180.0	16.0	60.0
Minimum	0.0	0.0	547.0	966.0	9.0	54.0	0.0	0.0	0.0
Maximum	400.0	409.0	810.6	1293.0	143.3	192.9	180.0	16.0	60.0
Sum	39,384.5	23,624.5	113,849.2	170,980.4	9432.3	19,185.4	12,100.6	1336.0	4380.0
Count	156.0	156.0	156.0	156.0	156.0	156.0	156.0	156.0	156.0

**Table 4:** N-estimator response of models.

Technique Used	Ensemble Approaches	Machine-Learning Methods	Ensemble Models	Optimum Estimator	R^2^-Value
Individual	-	DT	-	-	0.7623
	-	SVR	-	-	0.7923
Ensemble	Bagging	DT - Bagging	(10,20,30….200)	09	0.9206
		SVR - Bagging	(10,20,30….200)	12	0.9300
Ensemble	Boosting	DT - AdaBoost	(10,20,30….200)	05	0.9257
		SVR - AdaBoost	(10,20,30….200)	04	0.9005
Modified learner		RF	(10,20,30….200)	10	0.9388

**Table 5:** Statistical analysis.

Approaches Use	ML Methods	MAE	MSE	RMSE
Individual learner	DT	7.69	63.20	7.95
	SVR	5.69	55.20	7.43
Ensembling with AdaBoost	DT	3.59	20.70	4.55
	SVR	4.14	27.87	5.28
Ensembling with bagging	DT	3.22	21.52	4.64
	SVR	3.76	21.62	4.65
Ensemble model	RF	3.21	16.89	4.11

## Data Availability

The data used in this research has been properly cited and reported in the main text.

## Supplementary Materials

The following supporting information can be downloaded at: https://www.mdpi.com/article/10.3390/ma15103478/s1, Table S1: Parameters selected based on the literature review and the published data used in the prediction of fly ash slag-based concrete [62,63,64,65,66,67,68,69,70,71,72].
