# Supplementary material for: Prediction of Mechanical Properties of Fly-Ash/Slag-Based Geopolymer Concrete Using Ensemble and Non-Ensemble Machine-Learning Techniques
Source: Materials (Basel). 2022 May 12;15(10):3478. doi: 10.3390/ma15103478 (PMC9147112; doi:10.3390/ma15103478)
Supplement: Supplementary file 1 [file materials-15-03478-s001.zip › materials-1681119-supplementary.pdf]

Table S1: Parameters selected based on the literature review and the published data used in the prediction of fly ash slag-based concrete [62–72].

Fly ash (kg/m^3)	Fly ash composition(% weight)			GGBS (kg/m^3)	GGBS composition (% weight)			Aggregate (kg/m^3)		Alkaline activators (kg/m^3)		SP dosage (kg/m^3)	NaOH molarity	Temp. (^{o}C)	Compressive strength (MPa)	Ref.
	SiO_2	Al_2O_3	CaO		SiO_2	Al_2O_3	CaO	Fine	Coarse	NaOH	Na_2SiO_3					
252	60.11	26.53	4	108	34.06	20	32.6	774	1090.8	46.3	115.7	144	8	30	33.83	[72]
252	60.11	26.53	4	108	34.06	20	32.6	774	1090.8	51.4	128.6	144	8	30	36.19	
252	60.11	26.53	4	108	34.06	20	32.6	774	1090.8	56.6	141.4	144	8	30	31.11	
252	60.11	26.53	4	108	34.06	20	32.6	774	1090.8	61.7	154.3	144	8	30	25.71	
294	60.11	26.53	4	126	34.06	20	32.6	810.6	966	54	135	168	8	30	36.69	
294	60.11	26.53	4	126	34.06	20	32.6	810.6	966	60	150	168	8	30	38.16	
294	60.11	26.53	4	126	34.06	20	32.6	810.6	966	66	165	168	8	30	26.17	
294	60.11	26.53	4	126	34.06	20	32.6	810.6	966	72	180	168	8	30	25.11	
315	60.11	26.53	4	135	34.06	20	32.6	760.5	972	57.9	144.6	180	8	30	33.81	
315	60.11	26.53	4	135	34.06	20	32.6	760.5	972	64.3	160.7	180	8	30	38.96	
315	60.11	26.53	4	135	34.06	20	32.6	760.5	972	70.7	176.8	180	8	30	25.71	
315	60.11	26.53	4	135	34.06	20	32.6	760.5	972	77.1	192.9	180	8	30	20.76	
216	60.11	26.53	4	144	34.06	20	32.6	774	1090.8	46.3	115.7	144	8	30	42.32	
216	60.11	26.53	4	144	34.06	20	32.6	774	1090.8	51.4	128.6	144	8	30	47.92	
216	60.11	26.53	4	144	34.06	20	32.6	774	1090.8	56.6	141.4	144	8	30	46.87	
216	60.11	26.53	4	144	34.06	20	32.6	774	1090.8	61.7	154.3	144	8	30	43.38	
252	60.11	26.53	4	168	34.06	20	32.6	810.6	966	54	135	168	8	30	46.08	
252	60.11	26.53	4	168	34.06	20	32.6	810.6	966	60	150	168	8	30	51.22	
252	60.11	26.53	4	168	34.06	20	32.6	810.6	966	66	165	168	8	30	44.67	

252	60.11	26.53	4	168	34.06	20	32.6	810.6	966	72	180	168	8	30	46.57	
270	60.11	26.53	4	180	34.06	20	32.6	760.5	972	57.9	144.6	180	8	30	45.88	
270	60.11	26.53	4	180	34.06	20	32.6	760.5	972	64.3	160.7	180	8	30	50.69	
270	60.11	26.53	4	180	34.06	20	32.6	760.5	972	70.7	176.8	180	8	30	41.85	
270	60.11	26.53	4	180	34.06	20	32.6	760.5	972	77.1	192.9	180	8	30	45.68	[72]
180	60.11	26.53	4	180	34.06	20	32.6	774	1090.8	46.3	115.7	144	8	30	55.37	
180	60.11	26.53	4	180	34.06	20	32.6	774	1090.8	51.4	128.6	144	8	30	59.79	
180	60.11	26.53	4	180	34.06	20	32.6	774	1090.8	56.6	141.4	144	8	30	51.61	
180	60.11	26.53	4	180	34.06	20	32.6	774	1090.8	61.7	154.3	144	8	30	46.68	
210	60.11	26.53	4	210	34.06	20	32.6	810.6	966	54	135	168	8	30	56.86	
210	60.11	26.53	4	210	34.06	20	32.6	810.6	966	60	150	168	8	30	60.38	
210	60.11	26.53	4	210	34.06	20	32.6	810.6	966	66	165	168	8	30	46.48	
210	60.11	26.53	4	210	34.06	20	32.6	810.6	966	72	180	168	8	30	47.57	
225	60.11	26.53	4	225	34.06	20	32.6	760.5	972	57.9	144.6	180	8	30	48.91	
225	60.11	26.53	4	225	34.06	20	32.6	760.5	972	64.3	160.7	180	8	30	58.53	
225	60.11	26.53	4	225	34.06	20	32.6	760.5	972	70.7	176.8	180	8	30	48.45	
225	60.11	26.53	4	225	34.06	20	32.6	760.5	972	77.1	192.9	180	8	30	47.64	
252	60.11	26.53	4	108	34.06	20	32.6	774	1090.8	46.3	115.7	144	8	60	41.53	
252	60.11	26.53	4	108	34.06	20	32.6	774	1090.8	51.4	128.6	144	8	60	42.56	
252	60.11	26.53	4	108	34.06	20	32.6	774	1090.8	56.6	141.4	144	8	60	33.94	
252	60.11	26.53	4	108	34.06	20	32.6	774	1090.8	61.7	154.3	144	8	60	28.58	
294	60.11	26.53	4	126	34.06	20	32.6	810.6	966	54	135	168	8	60	40.41	
294	60.11	26.53	4	126	34.06	20	32.6	810.6	966	60	150	168	8	60	43.5	
294	60.11	26.53	4	126	34.06	20	32.6	810.6	966	66	165	168	8	60	31.24	
294	60.11	26.53	4	126	34.06	20	32.6	810.6	966	72	180	168	8	60	26.89	

315	60.11	26.53	4	135	34.06	20	32.6	760.5	972	57.9	144.6	180	8	60	38.63	
315	60.11	26.53	4	135	34.06	20	32.6	760.5	972	64.3	160.7	180	8	60	44.49	
315	60.11	26.53	4	135	34.06	20	32.6	760.5	972	70.7	176.8	180	8	60	35.2	
315	60.11	26.53	4	135	34.06	20	32.6	760.5	972	77.1	192.9	180	8	60	29.46	
216	60.11	26.53	4	144	34.06	20	32.6	774	1090.8	46.3	115.7	144	8	60	55.57	[72]
216	60.11	26.53	4	144	34.06	20	32.6	774	1090.8	51.4	128.6	144	8	60	57.37	
216	60.11	26.53	4	144	34.06	20	32.6	774	1090.8	56.6	141.4	144	8	60	53.39	
216	60.11	26.53	4	144	34.06	20	32.6	774	1090.8	61.7	154.3	144	8	60	51.22	
252	60.11	26.53	4	168	34.06	20	32.6	810.6	966	54	135	168	8	60	58.8	
252	60.11	26.53	4	168	34.06	20	32.6	810.6	966	60	150	168	8	60	62.29	
252	60.11	26.53	4	168	34.06	20	32.6	810.6	966	66	165	168	8	60	58.93	
252	60.11	26.53	4	168	34.06	20	32.6	810.6	966	72	180	168	8	60	56.64	
270	60.11	26.53	4	180	34.06	20	32.6	760.5	972	57.9	144.6	180	8	60	56.56	
270	60.11	26.53	4	180	34.06	20	32.6	760.5	972	64.3	160.7	180	8	60	61.3	
270	60.11	26.53	4	180	34.06	20	32.6	760.5	972	70.7	176.8	180	8	60	54.38	
270	60.11	26.53	4	180	34.06	20	32.6	760.5	972	77.1	192.9	180	8	60	49.83	
180	60.11	26.53	4	180	34.06	20	32.6	774	1090.8	46.3	115.7	144	8	60	61.96	
180	60.11	26.53	4	180	34.06	20	32.6	774	1090.8	51.4	128.6	144	8	60	65.26	
180	60.11	26.53	4	180	34.06	20	32.6	774	1090.8	56.6	141.4	144	8	60	53.39	
180	60.11	26.53	4	180	34.06	20	32.6	774	1090.8	61.7	154.3	144	8	60	52.4	
210	60.11	26.53	4	210	34.06	20	32.6	810.6	966	54	135	168	8	60	57.54	
210	60.11	26.53	4	210	34.06	20	32.6	810.6	966	60	150	168	8	60	64.28	
210	60.11	26.53	4	210	34.06	20	32.6	810.6	966	66	165	168	8	60	50.64	
210	60.11	26.53	4	210	34.06	20	32.6	810.6	966	72	180	168	8	60	49.44	
225	60.11	26.53	4	225	34.06	20	32.6	760.5	972	57.9	144.6	180	8	60	54.97	

225	60.11	26.53	4	225	34.06	20	32.6	760.5	972	64.3	160.7	180	8	60	61.9	
225	60.11	26.53	4	225	34.06	20	32.6	760.5	972	70.7	176.8	180	8	60	54.38	
225	60.11	26.53	4	225	34.06	20	32.6	760.5	972	77.1	192.9	180	8	60	53.79	
293	46	33	2.6	88	21	17	56.1	760	1005	143.3	71.7	0	6	20	37.4	[71]
293	46	33	2.6	88	21	17	56.1	760	1005	107.5	107.5	0	6	20	26.6	
293	46	33	2.6	88	21	17	56.1	760	1005	143.3	71.7	0	4	20	29.9	
293	46	33	2.6	88	21	17	56.1	760	1005	107.5	107.5	0	4	20	30.7	
344	46	33	2.6	34	21	17	56.1	760	1005	143.3	71.7	0	4	20	15.5	
316	46	33	2.6	63	21	17	56.1	760	1005	143.3	71.7	0	4	20	23	
293	46	33	2.6	88	21	17	56.1	760	1005	143.3	71.7	0	4	20	30.6	
253	46	33	2.6	108	21	17	56.1	760	1005	143.3	71.7	0	4	20	39.01	
272	46	33	2.6	108	21	17	56.1	760	1005	143.3	71.7	0	4	20	27.8	
253	46	33	2.6	126	21	17	56.1	760	1005	143.3	71.7	0	4	20	28	
237	63.53	27.4	1.26	158	34.26	11.32	38.34	547	1277	52	129	7.9	8	32	28.36	[70]
237	63.53	27.4	1.26	158	34.26	11.32	38.34	547	1277	52	129	7.9	4	32	34.84	
237	63.53	27.4	1.26	158	34.26	11.32	38.34	547	1277	52	129	7.9	4	32	37.24	
237	63.53	27.4	1.26	158	34.26	11.32	38.34	547	1277	52	129	7.9	4	32	33.2	
400	53.71	27.2	1.9	0	29.96	12.25	45.45	651	1209	45.7	114.3	0	14	23	25.6	
360	53.71	27.2	1.9	40	29.96	12.25	45.45	651	1209	45.7	114.3	0	14	23	38.3	
340	53.71	27.2	1.9	60	29.96	12.25	45.45	651	1209	45.7	114.3	0	14	23	46.6	
400	53.71	27.2	1.9	0	29.96	12.25	45.45	655.9	1218.1	40	100	6	14	23	32.5	
360	53.71	27.2	1.9	40	29.96	12.25	45.45	655.9	1218.1	40	100	6	14	23	33.3	
200	52.6	34.1	1.32	200	33.8	14.8	38.8	716	1074	9	56	0	0	0	31.7	[69]

200	52.6	34.1	1.32	200	33.8	14.8	38.8	712	1068	12	74	0	0	0	49.3	
200	52.6	34.1	1.32	200	33.8	14.8	38.8	708	1062	15	93	0	0	0	58.2	
200	52.6	34.1	1.32	200	33.8	14.8	38.8	712	1068	19	62	0	0	0	44.4	
200	52.6	34.1	1.32	200	33.8	14.8	38.8	708	1062	15	93	0	0	0	58.2	
200	52.6	34.1	1.32	200	33.8	14.8	38.8	712	1068	12	74	0	0	0	49.3	[69]
100	52.6	34.1	1.32	300	33.8	14.8	38.8	712	1068	12	74	0	0	0	52.1	
0	52.6	34.1	1.32	400	33.8	14.8	38.8	712	1068	12	74	0	0	0	63.1	
200	52.6	34.1	1.32	200	33.8	14.8	38.8	716	1074	15	93	0	0	0	65.7	
200	52.6	34.1	1.32	200	33.8	14.8	38.8	708	1062	15	93	0	0	0	58.2	
200	52.6	34.1	1.32	200	33.8	14.8	38.8	700	1050	15	93	0	0	0	42.3	
360	53.71	27.2	1.9	40	29.96	12.25	45.45	651	1209	45.7	114.3	0	14	22	40	[68]
320	53.71	27.2	1.9	80	29.96	12.25	45.45	651	1209	45.7	114.3	0	14	22	47	
360	53.71	27.2	1.9	40	29.96	12.25	45.45	651	1209	64	96	0	14	22	43	
320	53.71	27.2	1.9	80	29.96	12.25	45.45	651	1209	64	96	0	14	22	54	
400	53.71	27.2	1.9	0	29.96	12.25	45.45	658	1222	40	100	6	14	22	25	
360	53.71	27.2	1.9	40	29.96	12.25	45.45	655	1216	40	100	6	14	22	27	
320	53.71	27.2	1.9	80	29.96	12.25	45.45	655	1216	40	100	6	14	22	35	
400	53.71	27.2	1.9	0	29.96	12.25	45.45	658	1222	56	84	6	14	22	27	
360	53.71	27.2	1.9	40	29.96	12.25	45.45	655	1216	56	84	6	14	22	27	
320	53.71	27.2	1.9	80	29.96	12.25	45.45	655	1216	56	84	6	14	22	45	

360	53.24	26.42	3.65	40	36.77	13.56	37.6	644	1197	53	107	4	10	22	21.9	
340	53.24	26.42	3.65	60	36.77	13.56	37.6	646	1200	53	107	4	10	22	28	
320	53.24	26.42	3.65	80	36.77	13.56	37.6	648	1203	53	107	4	10	22	41.5	
300	53.24	26.42	3.65	100	36.77	13.56	37.6	650	1207	53	107	4	10	22	46	
280	53.24	26.42	3.65	120	36.77	13.56	37.6	652	1210	53	107	4	10	22	56.5	
340	53.24	26.42	3.65	60	36.77	13.56	37.6	646	1200	53	107	4	12	22	35	
320	53.24	26.42	3.65	80	36.77	13.56	37.6	648	1203	53	107	4	12	22	45	
300	53.24	26.42	3.65	100	36.77	13.56	37.6	658	1222	53	107	4	12	22	57	[67]
340	53.24	26.42	3.65	60	36.77	13.56	37.6	651	1209	47	93	4	10	22	30	
320	53.24	26.42	3.65	80	36.77	13.56	37.6	661	1227	47	93	4	10	22	40	
300	53.24	26.42	3.65	100	36.77	13.56	37.6	671	1246	47	93	4	10	22	48	
340	53.24	26.42	3.65	60	36.77	13.56	37.6	637	1184	64	96	4	10	22	28.5	
300	53.24	26.42	3.65	100	36.77	13.56	37.6	659	1223	46	114	4	10	22	45	
303.75	45.8	21.4	13.7	101.25	34.52	20.66	32.43	683	1269	81	81	4.05	8	0	10.5	[66]
303.75	45.8	21.4	13.7	101.25	34.52	20.66	32.43	683	1269	81	81	4.05	10	0	13	
303.75	45.8	21.4	13.7	101.25	34.52	20.66	32.43	683	1269	81	81	4.05	12	0	19	

303.75	45.8	21.4	13.7	101.25	34.52	20.66	32.43	683	1269	81	81	4.05	14	0	22	
303.75	45.8	21.4	13.7	101.25	34.52	20.66	32.43	683	1269	81	81	4.05	16	0	24	
303.75	45.8	21.4	13.7	101.25	34.52	20.66	32.43	683	1269	108	54	4.05	14	0	22.4	
303.75	45.8	21.4	13.7	101.25	34.52	20.66	32.43	683	1269	64.8	97.2	4.05	14	0	27	
303.75	45.8	21.4	13.7	101.25	34.52	20.66	32.43	683	1269	54	108	4.05	14	0	33	
303.75	45.8	21.4	13.7	101.25	34.52	20.66	32.43	683	1269	46.28	115.72	4.05	14	0	30	
303.75	45.8	21.4	13.7	101.25	34.52	20.66	32.43	683	1269	40.5	121.5	4.05	14	0	18.2	
303.75	45.8	21.4	13.7	101.25	34.52	20.66	32.43	683	1269	81	81	4.05	8	0	22.2	
303.75	45.8	21.4	13.7	101.25	34.52	20.66	32.43	683	1269	81	81	4.05	10	0	23.2	
303.75	45.8	21.4	13.7	101.25	34.52	20.66	32.43	683	1269	81	81	4.05	12	0	24	
303.75	45.8	21.4	13.7	101.25	34.52	20.66	32.43	683	1269	81	81	4.05	14	0	24.8	
303.75	45.8	21.4	13.7	101.25	34.52	20.66	32.43	683	1269	81	81	4.05	16	0	21.8	
303.75	45.8	21.4	13.7	101.25	34.52	20.66	32.43	683	1269	108	54	4.05	14	0	23.8	
303.75	45.8	21.4	13.7	101.25	34.52	20.66	32.43	683	1269	64.8	97.2	4.05	14	0	33.8	
303.75	45.8	21.4	13.7	101.25	34.52	20.66	32.43	683	1269	54	108	4.05	14	0	34.5	
303.75	45.8	21.4	13.7	101.25	34.52	20.66	32.43	683	1269	46.28	115.72	4.05	14	0	35	

303.75	45.8	21.4	13.7	101.25	34.52	20.66	32.43	683	1269	40.5	121.5	4.05	14	0	31.7	
0	0	0	0	400	37.73	14.42	37.34	810	990	57.1	143	8	12	25	89.6	[65]
0	0	0	0	400	37.73	14.42	37.34	810	990	57.1	143	12	12	25	89.2	
0	0	0	0	400	37.73	14.42	37.34	810	990	57.1	143	16	12	25	88.7	
0	0	0	0	400	37.73	14.42	37.34	810	990	57.1	143	20	12	25	86.11	
0	0	0	0	400	37.73	14.42	37.34	810	990	57.1	143	24	12	25	84.1	
204.5	65.6	28	1	204.5	30.61	16.24	34.48	554	1293	41	102	0	10	0	53.5	[63]
102	65.6	28	1	307	30.61	16.24	34.48	554	1293	41	102	0	10	0	55.5	
0	65.6	28	1	409	30.61	16.24	34.48	554	1293	41	102	0	10	0	58.6	
0	0	0	0	400	31.63	13.42	36.35	740	1110	12.9	82.5	0	0	22	34.6	[62]
0	0	0	0	400	31.63	13.42	36.35	785	1085	12.9	82.5	0	0	22	53.6	
0	0	0	0	400	31.63	13.42	36.35	790	1065	12.9	82.5	0	0	22	66.7	
